# Human Herpesvirus 6 (HHV-6) Encephalitis in a Non-Transplant Patient With Polymyositis

**DOI:** 10.7759/cureus.19314

**Published:** 2021-11-06

**Authors:** Vidya Baleguli, Young Min Cho, Jon Horn, Addison Parris

**Affiliations:** 1 Internal Medicine, Northeast Georgia Medical Center, Gainesville, USA; 2 Radiology, Northeast Georgia Medical Center, Gainesville, USA

**Keywords:** hhv6, ganciclovir, pcr, reactivation, encephalitis

## Abstract

Human herpesvirus 6 (HHV-6) was initially labeled as a human B lymphotropic virus because it was isolated in patients diagnosed with lymphoproliferative disorders. There are two variants of HHV-6: HHV-6A and HHV-6B. A considerable majority of recorded primary infections and reactivation events are primarily due to HHV-6B. We report a case of HHV-6 encephalitis reactivation in a 75-year-old Caucasian diabetic female with a past medical history of polymyositis treated with prednisone for a long time who presented with generalized weakness and drowsiness. She developed her symptoms after contact with her grandchildren, who recently had viral-like symptoms treated with antibiotics. Magnetic resonance imaging (MRI) of the brain without contrast showed 14 mm high transverse relaxation time (T2)/fluid-attenuated inversion recovery (FLAIR) signal intensity focus on the left temporal lobe, suspicious for primary versus metastatic neoplasm. Cerebrospinal fluid analysis found that protein concentration was 75 mg/dl, glucose concentration 55 mg/dl, white blood cell count was 22/mm3, with a lymphocytic predominance. Meningitis/encephalitis polymerase chain reaction (PCR) panel detected HHV-6. She was discharged after treatment with ganciclovir for 14 days. It is crucial to recognize HHV-6 infections in immunocompromised patients who present with a T2/FLAIR signal intensity focus in the left temporal lobe. In a hospital setting, rapid HHV-6 encephalitis testing is important to make a correct diagnosis to avoid any delay to prevent further morbidity and mortality.

## Introduction

Human herpesvirus 6 (HHV-6) was originally isolated in patients with a lymphoproliferative disorder, and it was labeled as a human B lymphotropic virus [1]. The two variants of HHV-6 are: HHV-6A and HHV-6B [2]. Most reported cases are from primary infections and reactivation due to HHV-6B.

HHV-6B infections are commonly described during the first three years of life during childhood. After primary infection, HHV-6B establishes latency like other herpes viral families. Roseola infantum, also known as exanthem subitum, is a well-known childhood disease caused by HHV6-B as a primary infection that resolves spontaneously. After the primary infection, the virus is known to replicate in the salivary glands and shed in the saliva. This is the recognized route of transmission for HHV-6 variant B strains. Subsequently, the virus remains latent in the monocytes and lymphocytes and endures in the tissues and cells at low levels. In adults, primary infection of HHV-6B is a rarely described entity [3].

Considering how rare the primary infection is in adults, most cases are considered to represent reactivation disease by HHV-6 [4]. In adults with HHV-6 seroconversion, mononucleosis-like symptoms with variable intensity and persistent lymphadenopathy have been described [3,5,6]. In rare instances, HHV-6 infection can be associated with different severity of encephalitis in immunocompetent patients. Clinical scenarios encountered can include alteration in mental status, seizures, psychosis, acute cerebellar ataxia, focal neurological signs, including but not limited to cranial nerve deficits or hemiparesis [7-10]. 

For treatment, foscarnet is known to be active against both HHV-6A and HHV-6B. On the other hand, ganciclovir is known to be active against HHV-6B, but some case reports described it as resistant to HHV-6A [11,12]. One study conducted in hematopoietic stem cell transplant recipients indicated that ganciclovir reasonably reduced HHV-6 in saliva compared with no therapy [13]. Even with these studies implying that specific antivirals may affect the replication of HHV-6, there have been no controlled clinical trials conducted to demonstrate such benefits in humans. Numerous circumstantial case reports and case series have advocated that there has been improvement in presumed HHV-6 encephalitis after treatment with either foscarnet or ganciclovir [7,14,15]. These conclusions require further validation in carefully controlled trials.

## Case presentation

A 75-year-old Caucasian female with a past medical history of polymyositis on a maintenance dose of prednisone of 10 mg daily, diabetes mellitus, status post right nephrectomy presented to the ED with complaints of generalized weakness and drowsiness. At baseline, the patient was minimally mobile due to active polymyositis. The patient was less responsive and confused with episodes of falling from her bedside commode. She denied having a cough, chest pain, shortness of breath, abdominal pain, nausea, vomiting, or diarrhea. In the last week, she had close contact with her grandchildren, who experienced a flu-like illness without rash treated with an antibiotic. 

On physical examination, her vitals were significant for elevated blood pressure of 174/82 mmHg, the temperature of 37.1 °C (98.7 °F), respiratory rate of 16 breaths per minute. Bilateral lower extremity swelling was present. On neurological exam, she was alert but not oriented on the place and time. Her Glasgow Coma Score (GCS) was 13. The patient showed decreased consciousness level, slowly followed commands, and slurred speech. Her reflexes were diminished: 2/4 in the bilateral biceps, triceps, brachioradialis, patellae, and Achilles. The rest of the exams were unremarkable without any focal neurological signs. Laboratory tests as noted in Table 1. The hepatitis panel was negative, and blood cultures showed no growth at 120 hours.

**Table 1:** Laboratory results

Laboratory Studies	Admission value	Reference Interval
Sodium	131 mEq/L	135-145 mEq/L
Potassium	3.1 mEq/L	3.6-5.2 mEq/L
Blood urea nitrogen (BUN)	71.0 mg/dL	7-20 mg/dL
Creatinine	2.71 mg/dL	0.74-1.35 mg/dL
Estimated glomerular filtration rate (eGFR)	16.1 mL/min/1.73m*2	>90 mg/mmol
Glucose	306 mg/dL	100-125 mg/dl
Troponin I	2.69 ng/mL	0-0.4 ng/mL
White blood cell (WBC)	5.1 K/uL	4.5-11 K/uL
Red blood cell (RBC)	3.61M/uL	4.7-6.1M/uL
Hemoglobin	9.8 g/dL	12-15.5 g/dL
Hematocrit	31.4 %	36-48%

Computed tomography (CT) brain without contrast showed scattered white matter hypodensities, which were nonspecific but compatible with chronic microvascular ischemic disease. 

**Figure 1:**
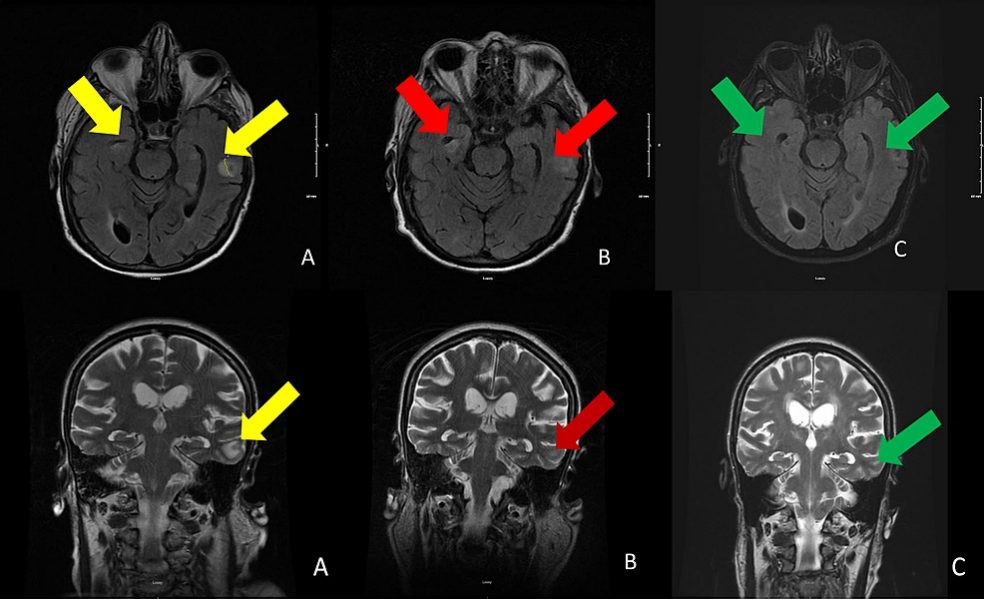
MRI of the brain without contrast, axial and coronal views A. MRI of the brain without contrast, on day five of admission, showed in the left temporal lobe with T2/fluid-attenuated inversion recovery (FLAIR) signal intensity 14 mm focus at the gray-white junction and right inferior temporal gyrus (yellow arrows). B. MRI without contrast on day 30 shows the subtle increased intensity of the right inferior temporal gyrus and decrease intensity in the left temporal (red arrows). C. MRI without contrast on day 176 shows improvement of the previous temporal and frontal enhancement (green arrows).

Further ischemic workup was deferred due to acute kidney injury in the setting of chronic kidney disease (CKD) stage 4. The patient had minimal responsiveness with a loop diuretic, and therefore she was started on hemodialysis for better volume management. MRI brain without contrast showed 14 mm high T2/fluid-attenuated inversion recovery (FLAIR) signal intensity focus in the left temporal lobe, suspicious for primary versus metastatic neoplasm (Figure 1). The neurologist was consulted and recommended a follow-up assessment with a gadolinium-enhanced MRI of the brain. 

CT chest/abdomen/pelvis with intravenous (IV) contrast did not find any malignancy or metastatic disease. Given the cortical location of intracranial lesions, she was started prophylactically on levetiracetam 250 mg daily. Electroencephalograms (EEG) showed generalized slowing and triphasic waves. MRI with contrast was deferred due to worsening kidney. With her history of chronic steroid therapy, a lumbar puncture (LP) was considered and showed protein 75 mg/dL (15-60 mg/dL), glucose 55 mg/dL (50-80 mg/dL), WBC 22/mm3 (0-8/mm3), 7RBC/mm3 (<1RBC/mm3), with a pleocytosis of lymphocytic predominance. Meningitis/encephalitis polymerase chain reaction (PCR) panel detected HHV-6. The patient was started on acyclovir 530 mg IV, and the infectious disease was consulted. Afterward, the patient was started on ganciclovir 75 mg IV as an induction dose for 14 days as ganciclovir has been shown in studies to be active against HHV6. Serologic testing also revealed a positive IgG 1:40 (reference <1:10) but negative IgM. Serum HHV-6 viral load was undetectable. 

Repeat MRI brain without contrast after 14 days of treatment with ganciclovir showed chronic microvascular-type ischemic disease and some T2 shine through in the left frontal area (Figure 1). The location of abnormality seen in the left temporal region in the subcortical white matter was less prominent. By this time, there was a significant improvement in her mental status. She was not placed on prophylaxis because there was no clear indication due to her marked symptomatic improvement and resolution of the area of abnormality in the left temporal region on the MRI. The patient has been discharged with her home regimen of prednisone 10 mg for polymyositis. Post-discharge at three-week outpatient follow-up, she was doing well without evidence of any further neurologic deterioration.

## Discussion

Because HHV-6 is a common infection in childhood, most adults express antibodies to the virus. For diagnosis of encephalitis, pneumonitis, and acute clinical disorders, isolation of the HHV-6 or detection of HHV-6 DNA in cerebrospinal fluid, brain or lung tissue, and respiratory secretions is required. In presumed HHV-6 encephalitis patients, the common symptoms are delirium, ataxia, amnesia, confusion, and seizure [16]. The pathophysiology is not understood, but multiple studies suggested a systemic reactivation of HHV-6 with high tropism for astrocytes in the hippocampus [17]. The typical MRI findings are hyperintensity on T2-weighted, FLAIR imaging, and diffusion-weighted imaging on bilateral medial temporal lobes, mainly affecting the hippocampus and amygdala [18]. However, MRI of the brain may be normal or demonstrate focal findings, such as augmentation in the temporal lobes [19]. 

There are no randomized controlled trials (RCT) showing the effectiveness of using antivirals to treat primary or viral reactivation caused by HHV-6. Several case reports have demonstrated successful treatment of HHV-6 associated encephalitis in bone marrow transplant recipients with either foscarnet or ganciclovir [11-13,20]. Unfortunately, there have been instances of reactivation and development of neurologic symptoms while these patients were receiving antiviral prophylaxis with these medications [14]. Despite this complication, due to the lack of other available therapies at present, it is reasonable to use one of these agents, alone or in combination, in immunocompromised patients with HHV 6 encephalitis. The existing data is less vibrant for the benefit of this therapy in immunocompetent patients, but treatment with one of these agents can be considered [14]. Numerous circumstantial case reports and case series have reported improvement in presumed HHV-6 encephalitis after treatment with either foscarnet or ganciclovir [8,14,15]. These conclusions require further validation in carefully controlled trials.

We suspect that infection in our patient may represent a reactivation due to chronic steroid exposure given her elevated IgG levels and negative IgM levels for HHV-6. HHV-6 viral load was likely not detected as levels were obtained following the initiation of ganciclovir. After 14 days of ganciclovir, the patient showed clinical and radiographic improvement, and we decided to hold the treatment and keep it in observation. She has been discharged on prednisone 10 mg for frequent flare-ups of polymyositis. There is not enough data to keep on prophylactic antiviral treatment. Upon a three-week outpatient follow-up, the patient continued to exhibit no further signs of neurologic decline. 

## Conclusions

As a clinician, it is vital to recognize HHV-6 infections in immunocompromised patients with a T2/FLAIR signal intensity focus in the left temporal lobe. Although HSV encephalitis is more commonly associated with high signal intensity on T2-weighted and T2 FLAIR images on MRI, it is crucial to consider HHV-6 infections as differential because a delay in treatment can increase morbidity and mortality. We were able to establish a diagnosis by using the meningitis/encephalitis PCR panel. We believe that having an available test tool for HHV-6 is very important. The indication of prevention in the post-treatment course is still unclear, and we think further investigation is crucial. 
